# (μ_2_-2-Meth­oxy­ethanol-κ^3^ *O* ^1^:*O* ^1^,*O* ^3^)(2-meth­oxy­ethanol-κ*O* ^1^)tris­(μ_2_-3,4,5,6-tetra­fluoro-*o*-phenyl­ene-κ^2^ *C* ^1^:*C* ^2^)trimercury(II)

**DOI:** 10.1107/S1600536814006898

**Published:** 2014-04-05

**Authors:** Raúl Castañeda, Sergiu Draguta, Andrey Yakovenko, Marina Fonari, Tatiana Timofeeva

**Affiliations:** a Department of Chemistry & Biology, New Mexico Highlands University, Diamond Ave, Las Vegas, NM 87701, USA; b X-ray Science Division, Argonne National Laboratory, 9700 S. Cass Avenue, Bldg 401 MS-16 Argonne, IL 60439, USA

## Abstract

In the title compound, [Hg_3_(C_6_F_4_)_3_(C_3_H_8_O_2_)_2_], two O atoms from one 2-meth­oxy­ethanol ligand and one O atom from the second 2-meth­oxy­ethanol ligand coordinate three Hg^II^ atoms [Hg—O = 2.765 (7)–2.890 (8) Å] in the trimeric organomercurial Lewis acid (*o*-C_6_F_4_Hg)_3_. The hy­droxy groups are involved in formation of intra- and inter­molecular O—H⋯O hydrogen bonds; the latter link two mol­ecules into centrosymmetric dimers. An extensive net of weak inter­molecular C—H⋯F inter­actions further consolidates the crystal packing.

## Related literature   

For the synthesis of trimeric perfluoro-*ortho*-phenyl­ene mercury and its use as a catalyst, see: Sartori & Golloch (1968 ▶) and Lee *et al.* (1999 ▶), respectively. For the properties of organomercurial anti­crowns, see: Taylor *et al.* (2007 ▶). For related crystal structures, see: Tikhonova *et al.* (2002 ▶, 2013 ▶).
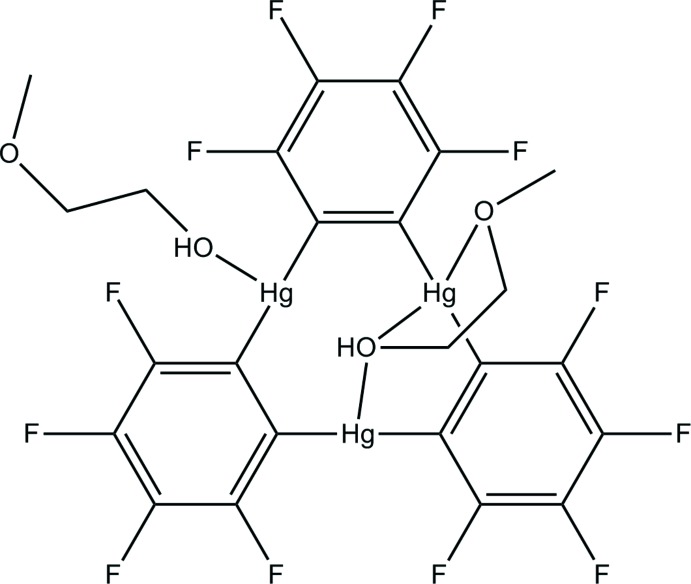


## Experimental   

### 

#### Crystal data  

[Hg_3_(C_6_F_4_)_3_(C_3_H_8_O_2_)_2_]
*M* *_r_* = 1198.14
Triclinic, 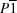

*a* = 10.170 (4) Å
*b* = 12.623 (5) Å
*c* = 12.962 (5) Å
α = 113.450 (5)°
β = 110.411 (5)°
γ = 92.795 (5)°
*V* = 1396.3 (9) Å^3^
*Z* = 2
Mo *K*α radiation
μ = 16.56 mm^−1^
*T* = 100 K
0.30 × 0.25 × 0.20 mm

#### Data collection  

Bruker APEXII CCD diffractometer
Absorption correction: multi-scan (*SADABS*; Sheldrick, 2003 ▶) *T*_min_ = 0.979, *T*_max_ = 0.984
16463 measured reflections
8430 independent reflections
4880 reflections with *I* > 2σ(*I*)
*R*_int_ = 0.090

#### Refinement  

*R*[*F* ^2^ > 2σ(*F* ^2^)] = 0.050
*wR*(*F* ^2^) = 0.101
*S* = 0.91
8430 reflections
386 parameters
H-atom parameters constrained
Δρ_max_ = 3.70 e Å^−3^
Δρ_min_ = −2.93 e Å^−3^

### 

Data collection: *APEX2* (Bruker, 2005 ▶); cell refinement: *SAINT* (Bruker, 2001 ▶); data reduction: *SAINT*; program(s) used to solve structure: *SHELXTL* (Sheldrick, 2008 ▶); program(s) used to refine structure: *SHELXTL*; molecular graphics: *SHELXTL*; software used to prepare material for publication: *SHELXTL*.

## Supplementary Material

Crystal structure: contains datablock(s) I. DOI: 10.1107/S1600536814006898/cv5445sup1.cif

Structure factors: contains datablock(s) I. DOI: 10.1107/S1600536814006898/cv5445Isup2.hkl

CCDC reference: 994110

Additional supporting information:  crystallographic information; 3D view; checkCIF report

## Figures and Tables

**Table 1:** Hydrogen-bond geometry (Å, °)

*D*—H⋯*A*	*D*—H	H⋯*A*	*D*⋯*A*	*D*—H⋯*A*
O2—H2⋯O3	0.84	2.04	2.771 (11)	145
O3—H3⋯O4^i^	0.84	1.86	2.694 (10)	171
C21—H21*B*⋯F6^ii^	0.99	2.52	3.480 (14)	162
C23—H23*A*⋯F10^iii^	0.99	2.48	3.109 (12)	121
C23—H23*B*⋯F11^iv^	0.99	2.47	3.301 (12)	141
C24—H24*B*⋯F5^ii^	0.98	2.54	3.339 (14)	138
C24—H24*C*⋯F2^v^	0.98	2.46	3.352 (15)	152
C19—H19*B*⋯F2^vi^	0.98	2.53	3.207 (13)	126

Symmetry codes: (i) 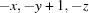; (ii) 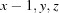; (iii) 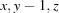; (iv) 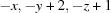; (v) 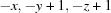; (vi) 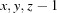.

**Fig. 1.**
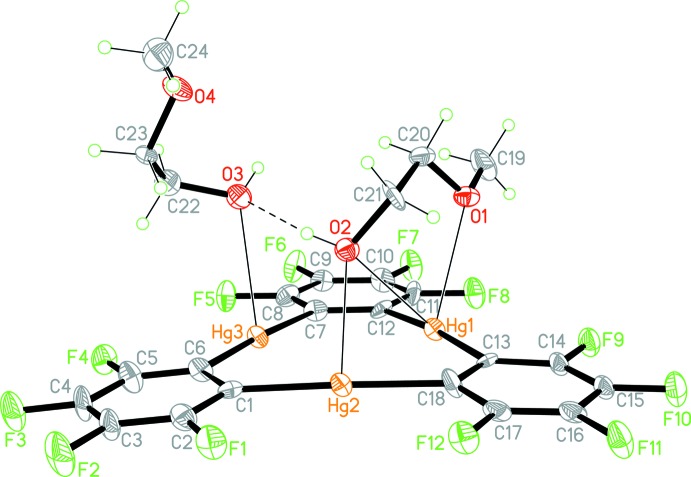
The molecular structure of the title compound showing the atomic numbering and 50% probability displacement ellipsoids. Dashed line denotes hydrogen bond.

**Fig. 2.**
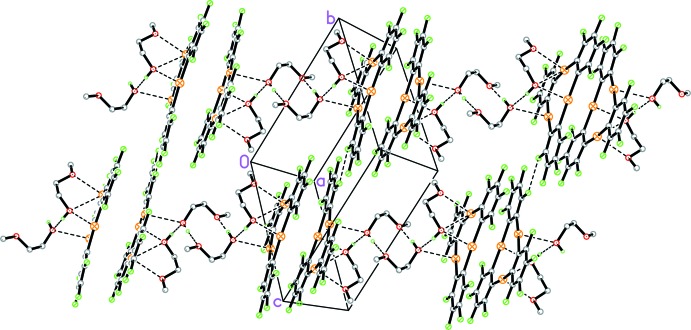
The crystal packing.

## supplementary crystallographic information

### 1. Comments

As an organomercurial compound trimeric perfluoro-*o*-phenyl­ene mercury (I) has numerous adducts with aldehydes, ketones, amides, nitriles, phospho­ramides, and sulfoxides (Taylor *et al.*, 2007). Formation of complexes involving I with oxygenated Lewis bases similar to 2-meth­oxy­ethanol (II) had been observed before (Tikhonova *et al.*, 2002). The studies of this type of coordination are important due the effect of I on the keto-enol tautomerism (Tikhonova *et al.*, 2013) as well on the activation of carbonyl compounds (Tikhonova *et al.*, 2002). Acting as Lewis acid I had proven catalyze the Diels-Alder reaction between thio­noester and cyclo­penta­diene (Lee *et al.*, 1999). Besides II is not a strong Lewis base it exhibits shorter Hg···O contacts than the sum of the van der Walls radii (Hg = 1.7-2 Å, O = 1.52 Å).

In the title compound (Fig. 1), the coordinating Hg···O contacts range from 2.765 (7) to 2.890 (8) Å. These short contacts are in the same range as those described for carbonyl compounds (Tikhonova *et al.*, 2002). The present complex [I·(II)_2_] has two II molecules per one I molecule. The hy­droxy groups are involved in formation of intra- and inter­molecular O—H···O hydrogen bonds (Table 1), and the latter ones link two complex molecules into centrosymmetric dimers. An extensive net of weak inter­molecular C—H···F inter­actions (Table 1) consolidate further the crystal packing. To the best of our knowledge this complex is the first coordination example of a non-cyclic ether with macrocycle I. That indicates a possibility of I to form complexes with non-cyclic ethers, like polyethyl­ene glycol derivatives.

### 2.1. Synthesis and crystallization

Trimeric perfluoro-o-phenyl­ene mercury (I) was synthesized according to the known procedure (Sartori & Golloch, 1968). The title compound [I·(II)_2_] was obtained by dissolving pure I in II, followed by slow evaporation of the solvent until single crystals were obtained.

### 2.2. Refinement

All hydrogen atoms were placed in the calculated positions with O—H = 0.84 Å, C—H = 0.98–0.99 Å and refined as riding, with *U*_iso_(H) = 1.2–1.5 *U*_eq_ of the parent atom.

### Crystal data

**Table:**

[Hg_3_(C_6_F_4_)_3_(C_3_H_8_O_2_)_2_]	*V* = 1396.3 (9) Å^3^
*M**_r_* = 1198.14	*Z* = 2
Triclinic, *P*1	*F*(000) = 1080
*a* = 10.170 (4) Å	*D*_x_ = 2.850 Mg m^−^^3^
*b* = 12.623 (5) Å	Mo *K*α radiation, λ = 0.71073 Å
*c* = 12.962 (5) Å	µ = 16.56 mm^−^^1^
α = 113.450 (5)°	*T* = 100 K
β = 110.411 (5)°	Prism, colourless
γ = 92.795 (5)°	0.30 × 0.25 × 0.20 mm

### Data collection

**Table:**

Bruker APEXII CCD diffractometer	8430 independent reflections
Radiation source: fine-focus sealed tube	4880 reflections with *I* > 2σ(*I*)
Graphite monochromator	*R*_int_ = 0.090
φ and ω scans	θ_max_ = 30.7°, θ_min_ = 4.4°
Absorption correction: multi-scan (*SADABS*; Sheldrick, 2003)	*h* = −14→14
*T*_min_ = 0.979, *T*_max_ = 0.984	*k* = −17→18
16463 measured reflections	*l* = −18→18

### Refinement

**Table:**

Refinement on *F*^2^	Primary atom site location: structure-invariant direct methods
Least-squares matrix: full	Secondary atom site location: difference Fourier map
*R*[*F*^2^ > 2σ(*F*^2^)] = 0.050	Hydrogen site location: inferred from neighbouring sites
*wR*(*F*^2^) = 0.101	H-atom parameters constrained
*S* = 0.91	*w* = 1/[σ^2^(*F*_o_^2^) + (0.0194*P*)^2^] where *P* = (*F*_o_^2^ + 2*F*_c_^2^)/3
8430 reflections	(Δ/σ)_max_ = 0.001
386 parameters	Δρ_max_ = 3.70 e Å^−^^3^
0 restraints	Δρ_min_ = −2.93 e Å^−^^3^

### Special details

**Table:**

Geometry. All e.s.d.'s (except the e.s.d. in the dihedral angle between two l.s. planes) are estimated using the full covariance matrix. The cell e.s.d.'s are taken into account individually in the estimation of e.s.d.'s in distances, angles and torsion angles; correlations between e.s.d.'s in cell parameters are only used when they are defined by crystal symmetry. An approximate (isotropic) treatment of cell e.s.d.'s is used for estimating e.s.d.'s involving l.s. planes.
Refinement. Refinement of *F*^2^ against ALL reflections. The weighted *R*-factor *wR* and goodness of fit *S* are based on *F*^2^, conventional *R*-factors *R* are based on *F*, with *F* set to zero for negative *F*^2^. The threshold expression of *F*^2^ > σ(*F*^2^) is used only for calculating *R*-factors(gt) *etc*. and is not relevant to the choice of reflections for refinement. *R*-factors based on *F*^2^ are statistically about twice as large as those based on *F*, and *R*- factors based on ALL data will be even larger.

### Fractional atomic coordinates and isotropic or equivalent isotropic displacement parameters (Å^2^)

**Table:**

	*x*	*y*	*z*	*U*_iso_*/*U*_eq_	
Hg1	0.34545 (4)	1.01863 (4)	0.29484 (4)	0.01824 (11)	
Hg2	0.22471 (4)	0.98504 (4)	0.51393 (4)	0.01800 (11)	
Hg3	0.41210 (4)	0.77523 (4)	0.36850 (4)	0.01824 (11)	
F1	0.1682 (7)	0.9075 (6)	0.7026 (6)	0.0307 (16)	
F2	0.2340 (9)	0.7301 (7)	0.7630 (7)	0.048 (2)	
F3	0.3741 (8)	0.5752 (7)	0.6553 (6)	0.0408 (19)	
F4	0.4452 (7)	0.5942 (6)	0.4792 (6)	0.0303 (16)	
F5	0.5756 (7)	0.6279 (6)	0.2121 (6)	0.0310 (17)	
F6	0.6681 (7)	0.6575 (6)	0.0508 (6)	0.0323 (17)	
F7	0.6287 (7)	0.8460 (6)	0.0030 (6)	0.0331 (17)	
F8	0.4924 (7)	1.0048 (6)	0.1120 (6)	0.0316 (17)	
F9	0.2731 (7)	1.2533 (6)	0.2847 (6)	0.0279 (16)	
F10	0.1551 (7)	1.4209 (6)	0.4062 (6)	0.0328 (17)	
F11	0.0669 (7)	1.3997 (6)	0.5718 (6)	0.0336 (18)	
F12	0.0954 (6)	1.2102 (6)	0.6197 (6)	0.0274 (16)	
O1	0.1057 (7)	0.9283 (7)	0.0754 (6)	0.0220 (17)	
O2	0.1230 (7)	0.8390 (7)	0.2563 (7)	0.0221 (17)	
H2	0.1125	0.7678	0.2434	0.033*	
O3	0.1619 (8)	0.6097 (7)	0.1593 (7)	0.0262 (18)	
H3	0.1431	0.5953	0.0860	0.039*	
O4	−0.1110 (8)	0.4592 (8)	0.0789 (6)	0.0261 (19)	
C1	0.2690 (11)	0.8440 (10)	0.5548 (9)	0.020 (2)	
C2	0.2353 (11)	0.8292 (11)	0.6427 (10)	0.025 (3)	
C3	0.2689 (14)	0.7414 (11)	0.6758 (10)	0.030 (3)	
C4	0.3408 (14)	0.6609 (11)	0.6203 (11)	0.030 (3)	
C5	0.3735 (12)	0.6747 (11)	0.5329 (11)	0.028 (3)	
C6	0.3446 (11)	0.7621 (10)	0.4967 (9)	0.018 (2)	
C7	0.4814 (11)	0.8017 (10)	0.2454 (9)	0.019 (2)	
C8	0.5518 (11)	0.7228 (11)	0.1895 (11)	0.026 (3)	
C9	0.6030 (11)	0.7385 (10)	0.1068 (10)	0.020 (2)	
C10	0.5808 (12)	0.8336 (11)	0.0835 (10)	0.025 (3)	
C11	0.5103 (11)	0.9119 (10)	0.1372 (11)	0.023 (3)	
C12	0.4586 (10)	0.8986 (9)	0.2192 (10)	0.018 (2)	
C13	0.2464 (10)	1.1399 (9)	0.3867 (9)	0.016 (2)	
C14	0.2302 (11)	1.2401 (10)	0.3691 (9)	0.0193 (17)	
C15	0.1701 (11)	1.3265 (10)	0.4274 (9)	0.0193 (17)	
C16	0.1251 (12)	1.3143 (11)	0.5125 (10)	0.024 (3)	
C17	0.1416 (11)	1.2187 (11)	0.5344 (9)	0.020 (2)	
C18	0.1981 (12)	1.1268 (11)	0.4728 (10)	0.024 (3)	
C19	0.1318 (14)	0.9019 (13)	−0.0342 (11)	0.036 (3)	
H19A	0.0444	0.8993	−0.0996	0.055*	
H19B	0.1587	0.8250	−0.0606	0.055*	
H19C	0.2100	0.9634	−0.0165	0.055*	
C20	0.0035 (11)	0.8364 (10)	0.0569 (10)	0.024 (3)	
H20A	0.0308	0.7599	0.0220	0.028*	
H20B	−0.0917	0.8328	−0.0025	0.028*	
C21	−0.0059 (12)	0.8555 (12)	0.1742 (11)	0.030 (3)	
H21A	−0.0190	0.9368	0.2149	0.036*	
H21B	−0.0907	0.7993	0.1573	0.036*	
C22	0.1456 (12)	0.4994 (11)	0.1684 (11)	0.029 (3)	
H22A	0.2294	0.5029	0.2386	0.034*	
H22B	0.1416	0.4333	0.0926	0.034*	
C23	0.0117 (11)	0.4786 (11)	0.1859 (9)	0.024 (3)	
H23A	0.0071	0.4087	0.2025	0.029*	
H23B	0.0120	0.5480	0.2576	0.029*	
C24	−0.2418 (12)	0.4380 (13)	0.0905 (12)	0.036 (3)	
H24A	−0.3226	0.4258	0.0152	0.055*	
H24B	−0.2444	0.5063	0.1603	0.055*	
H24C	−0.2490	0.3673	0.1040	0.055*	

### Atomic displacement parameters (Å^2^)

**Table:**

	*U*^11^	*U*^22^	*U*^33^	*U*^12^	*U*^13^	*U*^23^
Hg1	0.0181 (2)	0.0224 (3)	0.0180 (2)	0.00763 (18)	0.00949 (17)	0.0103 (2)
Hg2	0.0193 (2)	0.0209 (3)	0.0173 (2)	0.00739 (18)	0.00843 (17)	0.01047 (19)
Hg3	0.0176 (2)	0.0230 (3)	0.0186 (2)	0.00700 (18)	0.00915 (17)	0.0115 (2)
F1	0.047 (4)	0.031 (4)	0.024 (4)	0.019 (4)	0.025 (3)	0.012 (3)
F2	0.087 (6)	0.050 (5)	0.037 (5)	0.032 (5)	0.043 (4)	0.031 (4)
F3	0.071 (5)	0.041 (5)	0.037 (4)	0.029 (4)	0.036 (4)	0.029 (4)
F4	0.034 (4)	0.026 (4)	0.035 (4)	0.015 (3)	0.017 (3)	0.014 (3)
F5	0.036 (4)	0.028 (4)	0.046 (5)	0.017 (3)	0.025 (3)	0.024 (4)
F6	0.039 (4)	0.028 (4)	0.053 (5)	0.019 (3)	0.037 (4)	0.022 (4)
F7	0.042 (4)	0.043 (5)	0.042 (4)	0.022 (4)	0.034 (4)	0.030 (4)
F8	0.042 (4)	0.037 (5)	0.036 (4)	0.018 (4)	0.026 (3)	0.026 (4)
F9	0.040 (4)	0.026 (4)	0.026 (4)	0.013 (3)	0.016 (3)	0.016 (3)
F10	0.052 (4)	0.030 (4)	0.032 (4)	0.023 (4)	0.023 (3)	0.021 (4)
F11	0.051 (4)	0.039 (5)	0.029 (4)	0.029 (4)	0.024 (3)	0.023 (4)
F12	0.034 (4)	0.035 (4)	0.025 (4)	0.012 (3)	0.020 (3)	0.017 (3)
O1	0.021 (4)	0.023 (5)	0.017 (4)	0.004 (3)	0.006 (3)	0.005 (4)
O2	0.019 (4)	0.022 (5)	0.023 (4)	0.006 (3)	0.007 (3)	0.009 (4)
O3	0.034 (4)	0.028 (5)	0.018 (4)	0.004 (4)	0.012 (4)	0.011 (4)
O4	0.023 (4)	0.043 (6)	0.016 (4)	0.008 (4)	0.010 (3)	0.015 (4)
C1	0.021 (5)	0.016 (6)	0.014 (6)	−0.001 (5)	0.003 (5)	0.002 (5)
C2	0.021 (6)	0.028 (7)	0.025 (6)	0.007 (5)	0.008 (5)	0.014 (6)
C3	0.057 (8)	0.023 (7)	0.020 (6)	0.016 (6)	0.022 (6)	0.013 (6)
C4	0.054 (8)	0.030 (8)	0.032 (7)	0.020 (7)	0.030 (6)	0.024 (6)
C5	0.029 (6)	0.037 (8)	0.033 (7)	0.019 (6)	0.019 (6)	0.023 (7)
C6	0.022 (5)	0.025 (7)	0.014 (5)	0.010 (5)	0.015 (5)	0.007 (5)
C7	0.022 (6)	0.023 (7)	0.016 (6)	0.006 (5)	0.014 (5)	0.006 (5)
C8	0.021 (6)	0.024 (7)	0.030 (7)	0.000 (5)	0.011 (5)	0.009 (6)
C9	0.018 (5)	0.026 (7)	0.021 (6)	0.010 (5)	0.014 (5)	0.007 (5)
C10	0.026 (6)	0.036 (8)	0.022 (6)	0.007 (5)	0.019 (5)	0.015 (6)
C11	0.024 (6)	0.018 (7)	0.033 (7)	0.004 (5)	0.012 (5)	0.016 (6)
C12	0.010 (5)	0.011 (6)	0.025 (6)	0.000 (4)	0.007 (4)	0.000 (5)
C13	0.017 (5)	0.009 (6)	0.011 (5)	−0.002 (4)	0.002 (4)	−0.001 (4)
C14	0.028 (4)	0.021 (5)	0.014 (4)	0.009 (4)	0.009 (3)	0.011 (4)
C15	0.028 (4)	0.021 (5)	0.014 (4)	0.009 (4)	0.009 (3)	0.011 (4)
C16	0.029 (6)	0.028 (7)	0.018 (6)	0.016 (6)	0.013 (5)	0.008 (5)
C17	0.024 (6)	0.025 (7)	0.004 (5)	0.005 (5)	0.003 (4)	0.001 (5)
C18	0.033 (6)	0.029 (7)	0.023 (6)	0.018 (6)	0.014 (5)	0.019 (6)
C19	0.053 (8)	0.049 (9)	0.022 (7)	0.026 (7)	0.020 (6)	0.023 (7)
C20	0.020 (6)	0.021 (7)	0.020 (6)	0.004 (5)	0.003 (5)	0.003 (5)
C21	0.023 (6)	0.048 (9)	0.033 (7)	0.013 (6)	0.006 (5)	0.034 (7)
C22	0.033 (6)	0.026 (7)	0.025 (7)	0.010 (6)	0.012 (5)	0.008 (6)
C23	0.037 (7)	0.020 (7)	0.007 (5)	0.000 (5)	0.003 (5)	0.004 (5)
C24	0.030 (7)	0.042 (9)	0.043 (8)	0.005 (6)	0.016 (6)	0.024 (7)

### Geometric parameters (Å, º)

**Table:**

Hg1—C13	2.076 (10)	O2—C21	1.457 (12)
Hg1—C12	2.087 (10)	O3—C22	1.450 (14)
Hg1—O1	2.765 (7)	O4—C24	1.415 (13)
Hg1—O2	2.844 (9)	O4—C23	1.427 (12)
Hg2—C18	2.065 (11)	C1—C2	1.374 (15)
Hg2—C1	2.078 (11)	C1—C6	1.446 (14)
Hg2—O2	2.850 (8)	C2—C3	1.357 (15)
Hg3—C6	2.064 (10)	C3—C4	1.395 (15)
Hg3—C7	2.083 (10)	C4—C5	1.356 (15)
Hg3—O3	2.890 (8)	C5—C6	1.368 (15)
F1—C2	1.366 (12)	C7—C8	1.374 (14)
F2—C3	1.352 (13)	C7—C12	1.404 (15)
F3—C4	1.345 (13)	C8—C9	1.418 (15)
F4—C5	1.383 (12)	C9—C10	1.360 (16)
F5—C8	1.355 (13)	C10—C11	1.358 (15)
F6—C9	1.350 (11)	C11—C12	1.399 (15)
F7—C10	1.353 (11)	C13—C14	1.379 (14)
F8—C11	1.341 (12)	C13—C18	1.427 (14)
F9—C14	1.373 (11)	C14—C15	1.366 (14)
F10—C15	1.330 (12)	C15—C16	1.389 (15)
F11—C16	1.350 (12)	C16—C17	1.351 (16)
F12—C17	1.380 (12)	C17—C18	1.400 (14)
O1—C20	1.404 (13)	C20—C21	1.480 (15)
O1—C19	1.447 (12)	C22—C23	1.484 (15)
			
C13—Hg1—C12	174.4 (4)	C11—C10—F7	121.1 (11)
C18—Hg2—C1	175.0 (4)	C11—C10—C9	121.2 (10)
C6—Hg3—C7	175.8 (4)	F7—C10—C9	117.6 (10)
C20—O1—C19	111.1 (9)	F8—C11—C10	118.9 (10)
C24—O4—C23	112.5 (8)	F8—C11—C12	119.7 (9)
C2—C1—C6	117.7 (10)	C10—C11—C12	121.4 (10)
C2—C1—Hg2	121.4 (8)	C7—C12—C11	118.4 (9)
C6—C1—Hg2	120.8 (8)	C7—C12—Hg1	121.8 (8)
C3—C2—F1	117.8 (10)	C11—C12—Hg1	119.8 (8)
C3—C2—C1	122.9 (11)	C14—C13—C18	117.4 (9)
F1—C2—C1	119.3 (10)	C14—C13—Hg1	120.0 (8)
C2—C3—F2	121.0 (10)	C18—C13—Hg1	122.5 (8)
C2—C3—C4	120.4 (11)	C15—C14—F9	117.1 (9)
F2—C3—C4	118.6 (10)	C15—C14—C13	124.5 (10)
F3—C4—C5	123.8 (10)	F9—C14—C13	118.4 (9)
F3—C4—C3	119.3 (10)	F10—C15—C14	122.3 (10)
C5—C4—C3	116.9 (11)	F10—C15—C16	119.7 (9)
C4—C5—C6	125.6 (11)	C14—C15—C16	118.0 (10)
C4—C5—F4	116.0 (10)	C17—C16—F11	122.0 (10)
C6—C5—F4	118.4 (10)	C17—C16—C15	119.5 (10)
C5—C6—C1	116.5 (10)	F11—C16—C15	118.5 (10)
C5—C6—Hg3	120.4 (8)	C16—C17—F12	117.5 (9)
C1—C6—Hg3	123.1 (8)	C16—C17—C18	123.7 (10)
C8—C7—C12	119.4 (10)	F12—C17—C18	118.7 (10)
C8—C7—Hg3	119.1 (9)	C17—C18—C13	116.9 (10)
C12—C7—Hg3	121.5 (7)	C17—C18—Hg2	121.6 (8)
F5—C8—C7	121.3 (10)	C13—C18—Hg2	121.3 (8)
F5—C8—C9	117.7 (10)	O1—C20—C21	110.6 (10)
C7—C8—C9	121.0 (11)	O2—C21—C20	111.5 (9)
F6—C9—C10	122.0 (10)	O3—C22—C23	109.9 (9)
F6—C9—C8	119.4 (10)	O4—C23—C22	110.2 (8)
C10—C9—C8	118.6 (10)		

### Hydrogen-bond geometry (Å, º)

**Table:**

*D*—H···*A*	*D*—H	H···*A*	*D*···*A*	*D*—H···*A*
O2—H2···O3	0.84	2.04	2.771 (11)	145
O3—H3···O4^i^	0.84	1.86	2.694 (10)	171
C21—H21*B*···F6^ii^	0.99	2.52	3.480 (14)	162
C23—H23*A*···F10^iii^	0.99	2.48	3.109 (12)	121
C23—H23*B*···F11^iv^	0.99	2.47	3.301 (12)	141
C24—H24*B*···F5^ii^	0.98	2.54	3.339 (14)	138
C24—H24*C*···F2^v^	0.98	2.46	3.352 (15)	152
C19—H19*B*···F2^vi^	0.98	2.53	3.207 (13)	126

Symmetry codes: (i) −*x*, −*y*+1, −*z*; (ii) *x*−1, *y*, *z*; (iii) *x*, *y*−1, *z*; (iv) −*x*, −*y*+2, −*z*+1; (v) −*x*, −*y*+1, −*z*+1; (vi) *x*, *y*, *z*−1.
